# “For us here, we remind ourselves”: strategies and barriers to ART access and adherence among older Ugandans

**DOI:** 10.1186/s12889-019-6463-4

**Published:** 2019-01-31

**Authors:** Enid Schatz, Janet Seeley, Joel Negin, Helen A. Weiss, Grace Tumwekwase, Elizabeth Kabunga, Phiona Nalubega, Joseph Mugisha

**Affiliations:** 1 Department of Health Sciences, University of Missouri, Columbia, 535 Clark Hall, Columbia, MO 65211 USA; 2 Department of Global Health, London School of Hygiene and Tropical Medicine, London, UK; 3 Medical Research Council/Uganda Virus Research Institute and London School of Hygiene and Tropical Medicine, Uganda Research Unit, Entebbe, Uganda; 4 School of Public Health, University of Sydney, Sydney, Australia; 5 MRC Tropical Epidemiology Group, London School of Hygiene and Tropical Medicine, London, UK

**Keywords:** Aging, Antiretroviral adherence, Antiretroviral access, HIV care, Africa, Uganda

## Abstract

**Background:**

Very little is known about the barriers and facilitators to antiretroviral therapy (ART) access and adherence among older Africans. Most studies on ART have focused on individuals who are 15–49 years of age.

**Methods:**

We used in-depth interviews with 40 persons living with HIV, aged 50 to 96 years, who had either initiated ART (*n* = 26) or were waiting to initiate ART (*n* = 14), to explore barriers and facilitators to ART access and adherence in rural Uganda.

**Results:**

Guided by the Andersen Behavior Model, thematic content analysis highlighted 21 primary factors related to environment, patient and health behavior. Nine of the factors were common to both access and adherence, the remaining 12 were evenly split between access and adherence. Transportation costs, food insecurity, and healthcare workers’ knowledge, attitudes and behaviors were key barriers.

**Conclusions:**

These barriers were similar to those outlined for younger populations in other studies, but were compounded by age. Despite barriers, either due to the exceptional nature of HIV care or overreporting, both ART access and self-reported adherence were better than expected. Older persons living with HIV highlighted health care needs for non-HIV-related illnesses, suggesting while HIV care is adequate, care for the ailments of “old age” is lagging.

**Electronic supplementary material:**

The online version of this article (10.1186/s12889-019-6463-4) contains supplementary material, which is available to authorized users.

## Background

HIV infection is now a chronic condition due to widespread availability of antiretroviral therapy (ART), including in sub-Saharan Africa (SSA) [1–3]. As the epidemic continues, ART has led to a greater number of people living with, and thus aging with the infection, creating a growing proportion of people over 50 years old living with HIV [4, 5]. HIV infection has long been viewed as a condition of young people, for this reason nearly all policies, programs and data collection have focused on people aged 15–49 years [6, 7]. Because older persons may be individuals who have been on ART for a long time, and/or have a number of co-morbidities, their experience of ART may differ younger persons [8]. Little is known about the barriers and facilitators of ART access and adherence among older people [6, 9, 10]. Despite being neglected by HIV policies, strategies are critically needed for improving access, retention and adherence to ART for older persons living in SSA [10, 11].

The HIV treatment cascade, or care continuum, focuses on a number of steps from diagnosis to viral suppression. These steps include access to HIV testing; linkages to care; engaging in care; and achieving viral suppression. The United Nations (UN) has set a 90–90-90 target to have 90% of those individuals infected with HIV diagnosed, 90% of those living with HIV on ART, and 90% virally suppressed by 2020 [12, 13]. While there has been great improvement over the last decade in SSA; the percentages of people diagnosed, on ART, and reaching viral suppression fall short of these targets [14, 15]. The fact that there are few data on HIV testing, treatment and adherence among older people in SSA leads to an incomplete picture of HIV treatment. More data on barriers and facilitators to ACT access and adherence is required to reach these goals.

Interventions that will improve ART access and adherence for older people and other subgroups in resource-constrained settings need to address their specific barriers and facilitators to HIV care and treatment [9, 16]. Researchers in the US have found that depression and co-morbidities with other NCDs (non-communicable diseases; e.g., diabetes and hypertension) lower ART adherence and compromise HIV self-management among older adults [17–20]. On the other hand, self-efficacy, the level of confidence with which PLWH take their HIV medication, as well as feelings of social responsibility to family and community increase ART adherence [18, 19]. Older African-American women living with HIV in the US, have reported a desire for additional emotional and informational support from health care workers [21]. A major gap in the literature is on the experiences of OPLWH in SSA, research on older rural Ugandans’ suggests that access and adherence to ART are connected, with some barriers/facilitators for both either overlapping or reinforcing one another [22, 23].

The purpose of this qualitative study is identify the barriers and facilitators for accessing and adhering to ART. Drawing on the Andersen Behavioral Model (ABM) [24], the barriers and facilitators to be examined will include patient factors, health care environment factors and external factors. Our results can be used as a foundation for developing a sustainable and contextually appropriate intervention to improve ART access and adherence in Uganda, and more broadly in SSA.

## Methods

### Study design, sample & recruitment

Between September 2016 and March 2017 we conducted qualitative interviews with older persons (between 50 and 96 years) and key informants within Kalungu District in rural southwest Uganda. Participants were recruited using lists of older people accessing health and HIV services at three Level III health centers and the Medical Research Council/Uganda Virus Research Institute (MRC/UVRI) Health Clinic. Level III Health Centers exist within rural communities, covering about 20,000 people and are staffed by nurses. The MRC/UVRI Health Clinic is a designated government health facility.

Participants were stratified to include those not known to be infected, those who were HIV positive and were waiting to initiate ART, and older people living with HIV (OPLWH) on ART. We reported only interviews with those in the latter two categories: 26 OPLWH on ART and 14 waiting to initiated ART.

Changes in the Uganda ART treatment guidelines meant there were few older persons who had been diagnosed with HIV but were not yet receiving ART. Two of the health facilities did not have a record of HIV positive persons aged 50-plus years waiting to be initiated on ART. The majority in this category were recruited through the General Population Cohort and MRC/UVRI Health Clinic in Kyamulibwa, Kalungu district [25]. Although the WHO guidelines recommend “Test and Treat,” the roll out of this approach has been slow, largely because the available drug supply is limited. Those individuals who test positive, *are* generally started on Septrin prophylaxis, and remain on it even after starting ART, until the treating practitioner decides that the medication is no longer needed.

Ethical clearance was provided by the Uganda National Council for Science and Technology (HS2060); ethics approvals were also received from the Uganda Virus Research Institute and the University of Missouri.

### Data collection

The research team, which included experts in aging, HIV care, and qualitative methods, developed a semi-structured interview guide that explored themes related to older Ugandans’ ART access and adherence. The guide included: (1) participants’ experience of HIV and ART; (2) overall wellbeing and quality of life; (3) social support networks; and (4) engagement with the health system. Respondents receiving ART additionally self-reported their ART adherence, as well as barriers and facilitators to access, and treatment and medication adherence (see Additional file 1).

Participants’ age, sex, HIV and ART status were determined through clinic lists. Prior to interviews, all participants gave written/thumb printed consent. Interviews lasted 1–2 h, and were conducted by one of two members of the field team skilled in qualitative interviewing. All individual interviews were conducted at the participants’ homes. Interviews were audio recorded and field notes taken. We received written (or thumb printed and witnessed) informed consent from all participants.

### Conceptual framework

**Fig. 1:**
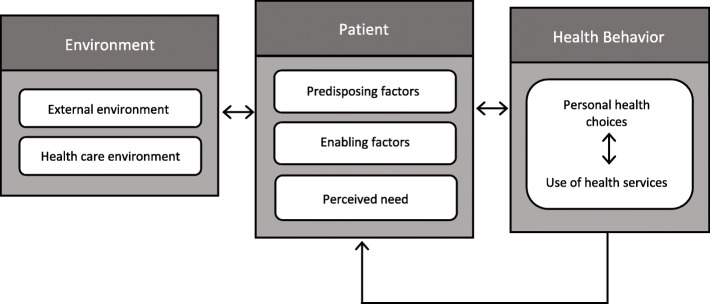
Image from Holtzman et al. (2015) adapted Andersen Model of Health Care Utilization (1995) (We received permissions to reproduce this figure)

Following Holtzman et al. [26], who worked in the United States, we use the Andersen Behavior Model (ABM) of Health Service Use to provide a theoretical framework for understanding older persons’ ART access and adherence in rural Uganda [24]. In the model, as adapted by Holtzman et al. [26] and shown in Fig. 1, there are seven domains: patient factors (predisposing, enabling, perceived need), health care environment factors (system, clinic, provider), and external environment factors. The inclusion of both environmental and patient factors create a stronger framework for building treatment interventions than other models [26]. We made use of data from both PLWH and health workers to understand health behaviors, and how environmental factors and patient factors influence and create feedback loops with ART access and adherence.

### Data analysis

The field team had regular debriefing sessions with the project manager and PI during the data collection period to discuss progress and outline impressions. Summaries of each audio recording, as well as full transcripts were completed for each interview. Adopting the thematic content analysis approach [27], we inductively developed themes and analyzed patterns emerging from the data. The research team (1) repeatedly read the summaries and transcripts to become familiar with the data, (2) coded the data by identifying key issues and recurrent themes related to ART access and adherence, and (3) organized the themes into categories. The codes and themes were based on participants’ accounts of barriers and facilitators to ART access and adherence. The data analysis was managed using NVivo version 11.

After coding the interviews, summary reports for each code related to ART access and adherence were produced in NVivo. The research team reviewed the ABM and mapped the themes and sub-themes to the domains outlined by Holtzman et al. (2015). The team adapted the model according to the emergent themes in this setting. Because of the similarity between the analyses and the applicability of the ABM, the coded data easily applied to the ABM domains. Barriers and facilitators to ART access and adherence were compared and assessed in conjunction with one another as many of them were similar. Ideas outlined in this paper were member-checked through a community feedback session with older persons and health workers from the communities in which the study took place. In addition, three of the authors (GT, EK, & PN) are members of the community and provided additional checks on the validity of themes.

## Results

In total, we interviewed 26 OPLWH on ART and 14 waiting to be initiated on ART (Table 1). There were no refusals, but from the original sampling lists of those on and waiting for ART, there was one individual who had died, two who were not known/could not be found within the community, and one who was too sick to interview. There were five individuals (4 waiting for ART and one already receiving ART) who did not disclose that they were living with HIV at the time of the interview and were not included in the analysis. One individual started ART, but had stopped taking the medication and was included in the study.

**Table 1:** Sample demographics

Characteristic	Total (*N* = 40)
Age (years)	
50–59	16 (40%)
60–69	14 (35%)
70–79	6 (15%)
80+	4 (10%)
Sex	
Male	22 (55%)
Female	18 (45%)
Initiated on ART	
ART	25 (62.5%)
Septrin only	14 (35%)
Stopped drug regime	1 (2.5%)

*ART* antiretroviral therapy

The median age of participants was 62 (range 50–96); 55% of respondents were male (*n* = 22), and about two-thirds were taking ART (63%; *n* = 25). Given the small numbers, we report age brackets rather than ages with respondent quotes.

Respondents identified 21 primary barriers/facilitators to ART access and adherence. Of these barriers and facilitators, 9 were common to both access and adherence, 6 were primarily associated with access and 6 were mainly discussed in connection to adherence (Table 2). Examples of each of the patient or individual level factors are in Table 3 (predisposing factors) and 4 (enabling factors), and environmental factors are in Table 5. Finally, Table 6 shows perceived needs at the patient level that highlight key respondent identified gaps between HIV care and other forms of care required in aging populations.

**Table 2:** Primary barriers/facilitators to ART access and adherence among OPLWH

Access	Adherence	Both
• Transportation (B) • Mobility (B) • Waiting times at clinic (B) • Health care workers’ disregard (B) • Appointment dates (F) • Free/low-cost of drugs (F)	• Reminder strategies (F) • Alcohol use (B) • Health literacy (B/F) • Travel (B) • Counseling (B/F) • ART as life saving (F)	• Poverty/Drought (Food/water security) (B) • Stigma (fear/shame) (B) • Disclosure & Kin/community support (B/F) • Depression (worry/loneliness) (B) • Medication Characteristics (B/F) • Health beliefs (F) • Symptoms (B) • Co-morbidities/NCDs (B) • Good care at health facility (F)

*ART* antiretroviral therapy, *OPLWH* Older people living with HIV, *NCDs* non-communicable diseases, *B* Barrier, *F* Facilitator

**Table 3:** Predisposing factors of ART access and adherence among OPLWH

ABM Domain	Barrier/Facilitator	Select quote	Access	Adherence
Predisposing factors	Health literacy	Reporting about the instructions she was given about her medicine for HIV, the old woman said; ‘they told me not to miss (take the drugs daily) they asked me the convenient time I would take and I told them at 9:00. Septrins are the ones that I take during the day and these others I take at night when I am going to sleep!’ *Woman, aged 60–69*		✔
	Alcohol use	‘It *(alcohol)* enable me get sleep and once I have taken some of it, I sleep like a dead body!’ *Man, discontinued ART, aged 80-plus*		✔
	Stigma	The bad thing is that when one is told that he is HIV infected, it scares him/puts him to shame. *Woman on ART, aged 60–69*	✔	✔
	Depression	Staying alone can prevent someone from not taking the drugs because she does not have anyone to assist with housework and by the time she finishes doing this and the other, she ends up forgetting. She concluded thus; ‘loneliness is a severe danger!’ She gave an example of an elderly community woman who was found dead in her house. She was staying all alone. *Woman on ART, aged 60–69*	✔	✔

*ART* antiretroviral therapy

### Predisposing factors

Psychosocial factors include economic and social circumstances that frame emotional and social meanings of health and contribute to overall life satisfaction [28]. The psychosocial factors frame the complexity of how health literacy, stigma, depression, and alcohol use are understood by respondents to affect ART utilization (Table 3) [28–31]. Health literacy and alcohol use were linked to adherence, while stigma and depression were seen as affecting both access and adherence.

The exceptionally high HIV-related health literacy among respondents living with HIV in terms of knowing when and how to take their medication was a key positive factor in adherence. Particularly given the level of education (most had very few years of formal school), this positive predisposing factor overshadowed many of the negative factors that respondents discussed. Even though the health literacy messages that respondents reported were not all correct, respondents claimed that they needed to take their ART with food. A woman on ART, aged 60–69, said that she was told that she should always eat something before taking her drugs because “HIV drugs are very strong.” She said the health worker never specified a type of food she should eat, but said she “should eat whatever I admire or what I eat and don’t vomit.” Many also said they were told to abstain from alcohol and sex, not just unprotected sex. A female respondent, aged 70–79, who had been on ART for nearly a decade, said that she “used to be a very good alcohol taker” but because of the “need to follow the health worker’s advice,” she reduced her drinking and eventually stopped completely. Perhaps due to feeling messages about sex and alcohol were overstated, some respondents related a wish to have additional counseling, and wanted reminders about what the dos/don’ts are related to ART. So while overall health literacy was high, there was a sense of insecurity about their knowledge, as well as (re)interpretation of information from providers which may have inadvertently increased challenges to adherence [32–34].

The majority of respondents related that they had reduced or stopped alcohol use themselves, primarily in response to recommendations as part of ART counseling, yet they noted that alcohol abuse was a key reason that older people (particularly men) may become non-adherent. One older woman on ART highlighted why adherence may be low for those who drink alcohol, “some people especially the alcohol takers, do not like to stop/or feel hard to abstain from drinking they therefore refuse to start the drugs” (*Woman on ART, aged 60–69).*

While stigma has lessened with the rollout of ART, and some respondents said things like, “the level at which this illness is now, I think every person is now used to it since it is no longer as scary as it used to be before, 20 or slightly less years ago. You would invite a friend to come and see an HIV/AIDS patient … They would grow very thin, with sores or rash all over yet people never understood what it really was.” (*Woman on ART, aged 60–69*) And yet, despite the shift of HIV from death-sentence to a chronic condition [3], stigma was presented as a barrier to both access and adherence. For example, being seen in a certain area of a clinic was said to identify one as living with HIV and on ART. Other respondents complained about a lack of confidentiality among health care workers, which reduced individuals’ desire to go to the clinic to access ART. One woman on ART, aged 50–59, at first said that “HIV is not shocking news in the community” but then went on to say, “Except there are some HIV infected persons who feel stigma especially when it comes to collecting drugs. They don’t want to be seen at the facility and what they do, a person gets a friend who is also on HIV drugs, he tells him that as you are going to collect your drugs help me and bring mine... There are the people who are on drugs and don’t want their partner to know about it. Such people when they come to the health facility they want to be weighed and bled for CD4 first when it comes to receiving drugs she wants a friend to receive it on her behalf.” In this vein, some respondents described hiding ART from others including spouses and sexual partners due to fear of repercussions from disclosure, increasing the likelihood of missing doses.

Feelings of social isolation and depression, while not clinically diagnosed, were often connected to and reflective of stigma related to both HIV and old age [35]. A man on ART, aged 60–69, described how loneliness could lead to defaulting, “even when a person lives alone at home, that can make him refuse taking the drugs because he has no one to remind him or to prepare something for him to eat before taking his drugs. He then says to himself, ‘Why do I not die and escape this miserable life?!’” The feelings of desolation led some respondents to say that they were less likely to go to their appointments for accessing ART and less likely to adhere to ART because they felt as if there was no reason to do so.

### Enabling/disabling factors

Resources and tools that boost ART access and adherence are enabling factors, and those which reduce access and adherence are disabling factors. Enabling/disabling factors to access included transportation and mobility; to adherence included reminder strategies, medications characteristics, and poverty/food insecurity; and to both ART access and adherence included travel and disclosure/social support (Table 4).

**Table 4:** Enabling/disabling factors of ART access and adherence among OPLWH

ABM Domain	Barrier/Facilitator	Select quote	Access	Adherence
Enabling factors	Transportation	‘I get challenged by money for transport especially when it comes to time for collecting my drugs from the {name} clinic. Sometime I miss appointment dates when I have to collect drugs due to lack of transport until I inform children to send me money.’ *Man, aged 60–69*	✔	
	Mobility	Reporting on the hardest part of accessing her treatment and making sure she takes her medication on time and when required, the old woman explained that nothing would have been hard for her but because of her painful feet, walking becomes hard though she tries to report as required when the appointed date has come. *Woman on ART, aged 70–79*	✔	
	Reminder strategies	‘I have a phone and a radio which enables me know the right time and when I happen to have gone somewhere, I cannot leave my drugs behind and I never miss a single day. Taking It (ART) is like taking food because without food one cannot remain strong and healthy.’ *Man on ART, aged 60–70*		✔
	Medication characteristics: Side effects	‘The tablets that MRC health workers gave me reduced the strength *(sexual strength/desire.)* I then asked them to delete me out of their books. *(To exclude his name out of those that had been enrolled for care.)* That the tablets had even caused me loss of appetite and did not know how I would survive without taking food.’ *Man, aged 80-plus*		✔
	Poverty: Food insecurity	‘They (the health workers) tell you thus; ‘you have to swallow the drugs after you have taken something (food.) However, there are times when I do not swallow the drugs having failed to get what to eat and I become confused!’ *Woman on ART, aged 60–70*		✔
	Travel	‘One time I lost a relative and went for the funeral at Gomba where I spent a week. The drugs got finished while I was still there. When I returned and went to the health facility, I apologized to the health workers for what had happened then they asked me to bring with me someone who I would send for my drugs in case I was unable to collect it due to unavoidable occurrences or when I have become sick and unable to go and pick it!’ *Woman on ART, aged 60–70*	✔	✔
	Disclosure: kin & community support	‘We (himself and wife) talk about collecting our HIV drugs at the facility and also remind each other when it comes to time of swallowing it.’ *Man on ART, aged 50–59* Those days I didn’t have any partner but these days after getting a partner, I do not collect drugs myself, I use a friend to collect it for me. I give her a record book and for her wait to go to the health facility at six months to check her CD4 and the viro-load. My friend who collects my drugs is also HIV positive and brings it when she has gone to collect her own.’ *Woman on ART, aged 50–59*	✔	✔

*ART* antiretroviral therapy, *OPLWH* Older People Living with HIV

Despite the relative proximity of our respondents to health clinics, a number of people had trouble accessing ART and general health care due to the cost and or distance that they had to walk to reach the clinic. As older persons, their mobility was often limited due to aging-related morbidities like fatigue and body pain, and/or symptoms of other co-morbidities, e.g. high blood pressure, diabetes, arthritis. The combination of lack of transportation and limited mobility limited access to care.

Almost all respondents identified that they were supposed to take their medication at a set time, and had set up reminders (radio program, clock on phone, breakfast/dinner time) as the time they took their medication every day. As one older man explained, ‘ART tablets are not sour and not many in number but only that they are big in size. Since my wife is also on HIV drugs, we remind each other. If the wife is swallowing her drugs, she asks me if I have already finished taking my dose. If I am going anywhere in the morning, the wife asks me if I have remembered to swallow my drugs before leaving the house *(Man on ART, aged 60–69).* Having a daily routine was helpful for many, but this meant that when they traveled (to visit children, go to funerals outside their community, etc.) that they often had challenges with adherence, either because they forgot their ART or did not take enough medication with them for the time they were gone. Other reminder strategies included kin who would ask each day if the older person had taken their medication—in most but not all cases the grandchildren or other kin knew that the medication was ART. Our respondents were less specific about how they remembered their appointment dates, which were often only once every two to three months if they were adherent, but the majority said they had not missed their appointments for receiving more ART. Travel outside of the area affected access, at times resulting in finishing ART and delaying refilling one’s supply.

The majority of our respondents were on a fixed dose combination, which only requires one pill per day, or two pills if they also continue to take Septrin. A woman on ART, aged 60–69, explained how she saw the importance of taking ART daily with her own eyes, “I swallow it regularly but there are some people who do not want to take it. They do exist. I had my second born, she fell sick with that disease (HIV infection). She had refused taking the drugs and by the time they brought her back to the village, she was very unconscious. She used to argue that taking daily drugs was not a normal practice. However, after being approached by health workers, she accepted and started receiving her treatment from Virus (MRC/UVRI clinic) and is now very healthy!” Few respondents complained about pill burdens, but some highlighted other medication characteristics including side effects and just being tired of taking medication daily as reasons for discontinuing ART or deciding not to return to the health facility.

Disclosure was a facilitator and barrier to both ART access and adherence in the OPLWH with whom we spoke. As noted above, many of our respondents reported having disclosed their status to family and friends. Having people know that they were living with HIV and on ART increased their ability to access care. These respondents felt they could ask for assistance and social support when needed. Disclosure improved adherence, as they were unashamed of taking out their medication, and received reminders to take their medication at the set times from family who knew their status. Thus, kin and family support, particularly when paired with disclosure, was a powerful resource for both social support and encouragement to take ART. As one 70–79 year old widow explained, “They [my grandchildren] ask me this, ‘What should we do now grandma? It is already time, take the tablets, put aside other issues!” Asked which tablets the grandchildren tell her to take, she quickly said; “Tablets for HIV. I disclosed to them my status because even the health worker advised me to inform them.” However, a number of respondents relayed that they purposefully did not disclose their status either to partners and/or to extended kin. Their reasoning often related to fear of repercussions, that the spouse/partner would leave them if their positive status was known. Having to conceal pills and appointments complicated access and adherence because pills were hidden outside of their houses, and each time they had to access the health facility for ART they had to invent an excuse.

Poverty as experienced through food insecurity was particularly problematic for adherence, since some respondents said they would skip doses if they did not have something to eat. A man, aged 80-plus, said that he sometimes misses doses “due to food problems” when he does not have food for supper. Hunger also made accessing ART more challenging, as it made traveling to the clinic both less desirable and more challenging, since the majority of respondents walked.

### Environmental factors

The external environment and the health care environment (outlined in Table 5) played critical roles in older people’s access to HIV care and adherence to ART. Prolonged dry weather was the primary factor related to physical, political and economic factors outside of the health care environment that mattered to our respondents. They complained about the ways that the lack of rainfall was affecting their livelihoods as subsistence and small-scale farmers and the pressure it was putting on their families in terms of food insecurity. An older women in her 70s said, “what can help maybe is having some little money so that we can get a supplement like a half cake or a chapatti from the shops during times of food scarcity from the garden because when there is no food, I completely do away with the taking of the drugs!” Older persons in this community are also caregivers to others, often their grandchildren. The need to take care of others, may at times take their focus, time, energy and resources away from their own health care needs and distract from ART access and adherence.

**Table 5:** Environmental factors (external and health care) of ART access and adherence among OPLWH

ABM Domain	Barrier/Facilitator	Select quote	Access	Adherence
External environment	Lack of rain and failed crops	About how life is, she mentioned that other than being disturbed by hypertension and food shortage, her life wouldn’t be bad. She told me that she gets well her HIV drugs and she adheres well. *Woman on ART, aged 60–69*		✔
Health care environment Clinic factors	Waiting time	He said whenever he would come to collect the drugs, he would reach at the MRC clinic in the morning, waited for drugs through the day and received it in the evening. It would cause him to go to the stall where they sell fried cassava to spend the money he had in the pocket or sometime to spend the whole day at the clinic hungry. What bothered him was the long line/ over waiting at the clinic which made him change and start accessing drugs at Kyamulibwa heath center (Government health facility). *Man on ART, aged 80-plus*	✔	
Provider factors	Counseling	Long time when their organization had just come, it used to come in the communities and sit with people to counsel them on what to do for example taking the medicine on time and taking it every day. Therefore if counselling is carried out on them it would help people to adhere well on the drugs. *Man on ART, aged 80-plus*		✔
	Disregard for older patients	Just a week ago when he went to Musanya’s health center, they cared for him very well and talking to him well (without barking at him). They took his weight and informed him his weight had increased by 2 k. This gave him courage to continue taking his drugs responsibly without skipping any day. *Man on ART, aged 60–69*	✔	✔
System Factors	Appointment times	She said that those days she used to get drugs for two months currently she gets drugs for three months. She said she has never missed to take her drugs. *Woman on ART, aged 60–69*	✔	✔
Health care environment	Cost/availability of drugs	He said that when he reaches at the health facility they weigh him and note /record his weight. He then waits for the basawo to call him to receive his drugs. He told me that he has never visited the facility and find that HIV drugs are out of stock. *Man on ART, aged 50–59*	✔	
	Good care	Regarding if she felt she received good care, she said; ‘yes, since they gave me my drugs in plenty for 2 consecutive months, I will return to the facility to collect more drugs in October 27th. They did not talk to me in an abusive way. I took all the tablets that were still remaining including the tablets I never swallowed due to lack of food. They then gave more drugs but emphasized that I should first complete the drugs that had remained before starting the newly given.’ *Woman on ART, aged 60–69*	✔	✔

*ART* antiretroviral therapy, *OPLWH* Older People Living with HIV

The health care environment plays a central role in both ART access and adherence. All but one facility in the area are government-supported health clinics, with services and medication provided and/or subsidized by the government. At these clinics, services were accessible without patient cost or need for insurance (as might be the case in other settings). The other facility is run by the Catholic diocese. There were fees related to ART to cover the cost to bring treatment from the urban center to the rural area. However, this fee was generally viewed as reasonable, and less costly than transportation expenditures needed to go to a more distant government health clinic. A man in his 50s who collects ART for himself, his wife and one of his children was asked if there were financial considerations to stay on ART, he explained, “Yes, they ask us to contribute a small amount for collecting the drugs. In my case here, I pay 5000 [shillings; about 1.35USD] each appointment for three of us!” He went on to say that he tries “by all means” to find the money but that if he does not have the money on his appointment date that, “the health workers do not like anyone who misses an appointment because of not having money, … so they go into an agreement with the health workers.” The drugs are issued to him, then he takes the money later.

Barriers to access included *clinic factors* such as waiting times, *provider factors* such as health care workers’ disregard for older patients, and *system factors* including appointment times and drug costs. In terms of adherence, provider counseling was a factor; and for both access and adherence, good care at the health facility (which spans across provider, clinic and system factors) was deemed important (Table 5).

All of the clinics had issues with long waiting times, which the older persons felt were particularly difficult for them because it usually meant that they spent the day without food or water. Some respondents said that waiting times were shorter for ART than for other types of health appointments. One older man had switched facilities and was pleased with the government health facility where he now accesses ART. He said, “The line there is not long. When you reach there, the health workers give you the drugs and you return home in time.” When waiting times existed, it was connected to a lack of appointment scheduling. Although most of our respondents seemed to have knowledge of a date they were supposed to return to collect ART, there were no appointment times. Individuals were seen on a first-come first-served basis. While this seemed to work well enough for ART pick up, if OPLWH needed to see a health worker for any symptoms, health issues, or other needs, they were faced with long waiting times unless they arrived very early in the morning. As we have found in other work from this area, older persons have concerns about the ways they are seen and treated by health care workers. They worry about being shouted at and scolded for not following directions properly [22].

ART is free of charge, thus, drug cost was not a barrier to access or adherence for any of the respondents. The Ugandan government provides HIV-related drugs as they are on the government’s *essential drug list*. Because of international support for ART in Uganda, and a strong NGO presence in the area, stock-outs of ART were very rare. Only one or two respondents noted having to return for additional ART sooner (within a month rather than two or three months) for their next appointment due to limited drug supplies. None of our respondents reported being unable to acquire ART at their appointments.

Respondents felt they adhered well when they had “good counseling,” but many wished for continued counseling, which seemed lacking once individuals were receiving ART. The desire for additional counseling may have been related to mental health concerns. One older woman who felt good about her experience with ART explained that when she has discouraging thoughts or worry, “regular contacts with [health workers] help; they tell me to avoid thoughts.” Finally, “good care” at the health facility contributed both to individuals’ willingness to return to the facility to access ART, and their desire to follow the instructions of their health providers and adhere well.

### Perceived needs

*Perceived needs* capture issues that OPLWH see as lacking; the term perceived does not imply that these needs are not “real,” but rather that the needs were observed and expressed by the OPLWH themselves (Table 6). Our respondents’ perceived needs included their health care beliefs and their values around health. The OPLWH talked about health beliefs, symptoms and issues related to co-morbidities (usually NCDs) as connected to both access and adherence. One of the key health beliefs that increased the likelihood to access ART on time and adhere to ART as prescribed was that ART was lifesaving. Respondents had seen people die of AIDS-related illnesses earlier in the epidemic and related stories of how ART revived them, and allowed them to live well. A woman, aged 70–79, commented that she looks at ART as “her mother, father and everything in her life.”

**Table 6:** Perceived needs in relation to ART access and adherence among OPLWH

ABM Domain	Barrier/Facilitator	Select quote	Access	Adherence
Perceived needs	Health Beliefs	“I at one time tried swallow those tablets when I had not eaten anything and I felt as if I had become drunk! The drugs are very strong and I almost collapsed. Even when you take it having taken a little food, you still feel as if something wrong has happened to you! You feel weak and having no strength to do any work. However, after about 2 h, you begin coming back to normal. The drugs are very strong.” *Woman on ART, aged 60–69*	✔	✔
	Symptoms	Narrating about the most challenging symptoms/illnesses, the older woman said; ‘that problem that makes the leg borne as if bursting plus the excess heat inside the feet which extends right up to the private parts! I would rather suffer from HIV/AIDS but when I combine failure to feed on a good diet and suffering from all those illnesses; I then live a complete miserable life!’ *Woman on ART, aged 60–69*	✔	✔
	NCDs/Co-morbidities	Regarding if taking her medication was a problem, she said it was not because she freely took both her ART and the other for hypertension. It would have been hard for her to get the money to buy the tablets for hypertension by the time she was staying at her son’s home because it cost 7000/=. Her son is the one who paid for it when the wife of the land lord brought it. She takes 1 tablet daily. *Woman on ART, aged 60–69*	✔	✔
	Mental health	“One day I wished to put my life to an end through committing suicide but I failed” he said. I was very much concerned when he talked about this. He went on to say there was one day I thought about what I was going through, I used to walk but due to dislocation it is impossible to walk. I had a lot of pain; I used to walk to the toilet but I cannot not do that anymore, I wished that committing suicide would be the right solution, I swallowed the batteries of a watch hoping I was going to die but it failed to kill me. *Man on ART, aged 80-plus*	✔	✔

*ART* antiretroviral therapy, *OPLWH* Older People Living with HIV, *NCDs* non-communicable diseases

The majority of perceived needs relate to not being able to access care or adhere as well, or get the same type of “life saving” results, from other medications and health services as received for HIV. A number of respondents spoke about the difference between accessing ART and medication for chronic conditions, particularly hypertension. The latter often required paying for drugs, or finding out that some of the prescribed drugs were not in stock and needed to be bought elsewhere. One woman in her 60s explained how she was able to take both hypertension medication and her ART, but that unlike her ART that are free, “it is difficult to get the money to buy the tablets for hypertension.” When she was diagnosed, she was staying at her son’s home, and he paid the 7000 Uganda shillings [~2USD] for the tablets. Mental health, whether connected to depression and social isolation discussed above, related to dementia of aging, or other mental health issues, is an area that has received less attention. The most extreme case was man in his 80s who was despondent and in pain, so attempted suicide by swallowing batteries (see quote in Table 6); another woman in her 60s, who was waiting to be put on ART said that although she knows that suicide is not the answer, that she “lives in hardship” and “no one helps or supports” her. Given these circumstances, it was not surprising that there were perceived (and real) mental health needs, which must be addressed as a component of general health to improve overall quality of life among older persons [36].

## Discussion

The barriers to ART access among older Ugandans, included transportation, mobility, waiting times at the clinic, health care workers’ disregard, and lack of appointment times. The low cost of drugs facilitated access. In this setting, older persons have limited access to cash, as there are no social grants for older persons. This fact combined with their mobility which is often limited by age and health, means that it is harder to get to the clinic. Previous research on treatment access for the aging population in this study area has highlighted transport and treatment costs as barriers to care [22]. Older persons also worry about the ways in which ageism, related to HIV being a disease for young people, but also more general disregard, makes them feel that the clinic is not for them. Healthcare workers’ knowledge, attitudes and behaviors also influenced whether PLWH, of all ages, access HIV care [37]. The health care system works on a first come first serve basis, except for critical cases. The lack of appointment times means that it is often hard on older persons to sit for long periods of time without food or water [22, 37, 38]. Despite these barriers to access, few of our participants mentioned missing an appointment or not being able to access ART at their assigned time.

There was a separate set of barriers and facilitators to ART adherence. High levels of health literacy, the use of a variety of reminder strategies and viewing ART as life-saving all contributed to adherence. Alcohol use, travel and a sense of the need for additional counseling acted as barriers to ART adherence. Perhaps for older persons in particular, who had to care for their adult children and see them die from HIV/AIDS related causes, the ability to live on ART is particularly poignant [39]. Older persons made use of reminder strategies that used time and the radio. Spouses, grandchildren, and adult children checked up on them to make sure they took their daily dose of ART. This type of reminder may have made them feel they are cared about and connected to family, which is an important component of wellbeing for older adults [40]. For those without family, the sense of loneliness and wondering what there was to live for was a barrier to adherence [41]. Among older men in particular, alcohol may increase feelings of hopelessness and the likelihood to default [42, 43]. While these barriers to adherence are not insignificant, for the most part, the 26 respondents on ART, self-reported strongly adherent behavior – saying that they only missed a dose occasionally.

While there were separate barriers to access and adherence, the majority of themes raised in the interviews affected both, highlighting the interconnectedness of these two features of the HIV treatment cascade. Barriers to access and adherence included food and water insecurity, stigma, depression, health beliefs, symptoms and co-morbidities. Several US studies of older adults have similarly found that ART adherence and HIV self-management are compromised by both depression and co-morbidities with other non-communicable diseases (e.g., diabetes and hypertension) [17–20], or by the intersection of stigma with aging and other marginizing factors [30, 31, 35]. Because of the complexity of health issues experienced by older persons living with HIV, in both our study and a study in the US, participants expressed a difficulty managing their overall health, and decerning what symptoms were HIV-related and which were related to aging [44]. Three key aspects that facilitated both access and adherence were the ease and regularity of the ART regime, disclosure resulting in kin and/or community support, and good care at the clinic. These themes occuring in work among older persons in the US as well, where social connection to family and the community, as well as self-efficacy increases ART adherence [18, 19].

Study limitations include having relatively few interviews with individuals who had defaulted or were having trouble accessing ART. It is likely that these individuals exist in the area, but are less likely to be engaged in care at all and so are less likely to be identified through clinic lists (our primary sampling frame). Health care workers with whom we spoke acknowledged that there are OPLWH in the community who are not adherent. An intervention involving a lay counselor with knowledge about older persons’ unique health, social and psychosocial needs who visits OPLWH in their homes may allow for referrals to both social and health services when needed.

## Conclusion

The interconnections between ART access and adherence are many, as is evidenced by the number of themes that related to both aspects of HIV care. The interrelatedness stems from economic, cultural and personal factors that affect both the ability and desire to access ART and the ability and desire to adhere to ART on a daily basis [26, 45, 46]. The importance of assessing these in conjunction with one another and in a way that brings to the surface the interaction between barriers and facilitators are key to understanding the experiences of older Africans living with HIV and on ART. As is seen in the examples above, access is necessary for adherence to be viable, but there are also ways that certain facilitators – e.g. money for transport, can improve the likelihood of both access and adherence.

HIV services and care are much better resourced and administered than most other health services in SSA [47, 48]. Our respondents reported high levels of both ART access and adherence despite existing barriers. There are no studies of older persons in SSA to assess the concordance between self-reported adherence and viral load. Extant work on younger populations, however, shows that while self-reported behavior generally overstates adherence, higher self-reported adherence does correspond with a higher likelihood of being virally suppressed [49]. Taking advantage of and building upon the apparent competency in self-management and self-efficacy of this population in adhering to ART could further enhance adherence [18, 19, 50, 51]. And yet, even with high levels of self-reported adherence and self-efficacy, it would be wrong to conclude that HIV diagnosis and treatment, or health care more generally, are reaching older Ugandans sufficiently.

There are HIV vertical programs that ensure drug supply even to rural clinics, as well as health workers trained in diagnosis, counseling, testing, and monitoring adherence [52]. While challenges remain in HIV service delivery, the attention that has gone into HIV care is exceptional. Further, the area where we are working, similar to other settings in southern and central Uganda, has a strong NGO presence, Uganda CARES, that provides ART to the area, as well as a concentration of well distributed government health services. The care provision in this area is important to document, but also means that the barriers outlined above are likely to be even greater in areas with a poorer drug supply and fewer available services. In all likelihood, this means if anything the barriers outlined are understated compared to areas with fewer available services. Given the rural setting and ongoing drought in this area, some of the barriers are likely to be either different from or exacerbated compared to other settings, limiting the generalizability of our findings.

The ABM category of perceived needs importantly brought attention to older persons’ desires for HIV-like services for their non-HIV-related ailments. While this paper focused on ART access and adherence, it is also important to note that ART is a special situation due to the NGO and international donor support. There is a particular need to increase knowledge and treatment for diseases of aging, whether frailty or non-communicable diseases that are seen more commonly among older persons than younger persons living with HIV as co-morbidities [8]. Other illnesses suffered by older persons are generally subject to poorer knowledge on the part of practitioners, as well as more breakdowns in the supply chain. Yet, it is well known that those aging with HIV are likely to have co-morbidities, and that NCDs (hypertension, diabetes, arthritis) are common and becoming more prevalent in this region [53, 54]. Thus, health care delivery in these settings must also address the growing burden of NCDs, as well as the likely increase in multi-morbidity as individuals age on ART. Interventions that address ART access and adherence must, therefore, be cognizant of both the illnesses of “old age” *and* HIV on older persons’ health and health care needs as the HIV and NCD epidemics converge.

## Additional file

Additional file 1: Qualitative Individual Interview Guide for Leave No One Behind Study. (DOCX 17 kb)

## Abbreviations

ABM: Andersen Behavioral Model
ART: Antiretroviral therapy
HIV/AIDS: Human immunodeficiency virus/Acquired immunodeficiency syndrome
MRC/UVRI: Medical Research Council/Uganda Virus Research Institute
NCD: Non-communicable diseases
OPLWH: Older persons living with HIV
PLWH: People living with HIV
SSA: Sub-Saharan Africa
UN: United Nations
