# Supplementary material for: “For us here, we remind ourselves”: strategies and barriers to ART access and adherence among older Ugandans
Source: BMC Public Health. 2019 Jan 31;19:131. doi: 10.1186/s12889-019-6463-4 (PMC6357505; doi:10.1186/s12889-019-6463-4)
Supplement: Supplementary file 1 — Qualitative Individual Interview Guide for Leave No One Behind Study. (DOCX 17 kb) [file 12889_2019_6463_MOESM1_ESM.docx]

***Leave No One Behind Study***

*Qualitative Interview Guide for Individual Interviews with older persons*

Questions were tailored depending on HIV and ART status as disclosed by respondent

1. **EXPERIENCE OF HIV & OVERALL WELLBEING/QUALITY OF LIFE**

- identifiers (IDNO, age, sex, religion, education level, marital status, income sources, household composition and relationship with each of the members the respondent stays with)
- How is your health these days? How is your physical health? What makes you feel healthy? What makes you feel sick?
- How is your mental health? What makes you feel healthy? What makes you feel sick?
- What symptoms or illness have you experienced in the last 30 days? What symptoms or illness are the most challenging? Why? Where/how do you seek help when you have these symptoms/illness?
- What things affect your daily wellbeing? (e.g., sleep, access to physical care, food security, medication)
- How would you rate your quality of life (poor, fair, good, very good, excellent)? What makes your life this rating? What would you need to improve your quality of life?

1. **SOCIAL SUPPORT/ECONOMIC SUPPORT**

- Who do you go to when you need social support? (probe for characteristics of person/group)
- How easy is it for you to reach that person (by phone, visit, transport)? Is that person/people/organization/group usually available?
- How much/what kind of help do you mainly need/seek from this person/group? Do you feel like this person/group is usually able to help to your satisfaction?
- When you receive social support, do you reciprocate in any way? If so, how and why? If not, why not?
- What barriers do you face to maintaining relationships and accessing social support?
- What facilitates your ability to maintain relationships and access social support?
- What are things you do to make you feel connected to your community/family?
- What are things that you wish you had in your life to make you feel more connected to the community/family?
- A lot of older people from this community feel socially isolated, do you feel that way?
- Would you like to meet with other people your age on a regular basis?
- Would you like if an older person visited your home regularly to check on you?
- What kinds of interactions would be the most beneficial?
- What economic challenges do you have currently? Where/how do you get seek help when you have economic challenges?

1. **TREATMENT ACCESS & ADHERENCE**

- How did you come to know you were HIV-positive?
- Why did you decide to be tested?
- Are you on ART?
- If you are on ART, When did you begin ART/ how many years have you been on ART/how old were you when you started?
- How did you come to be on ART/who, and where were you initiated? Who first told you about ART
- Have you had any experiences of stigma because of being HIV-positive? Because of being on ART? Tell me more about times when you have felt this way.
- Have you felt socially isolated because of being HIV-positive? Because of being on ART? Tell me more about times when you have felt this way.
- Are you on any medication prescribed by a health worker (doctor nurse etc.)? Is getting your medication a problem? (probe for transportation to access, supply at clinic, treatment upon pickup) Why/how?
- Is taking your medication a problem? (probe for memory, water/food to take with medicine, diet) Why/how?
- Are you also on any alternative medication? Is getting your medication a problem? Is taking your medication a problem? Why/how?
- What instructions did you get about your medicine for HIV? (probe for diet, regime, need for adherence)
- How do you make sure that you take your medicine on time and when required?
- What is life like on treatment? Are there reasons why someone would NOT want to be on treatment? Does this change as people get older?
- What is the hardest part accessing your treatments, and making sure you take your medicine on time and when required?
- Are there any financial considerations to staying on treatment?
- What do you think could help you? Do you think having a group or individual who helped you remember would make it easier? Why/why not?
- How does the community treat people with your disease? People on treatment? Is it different for older persons than it would be for younger adults?
- Who helps or supports people to get diagnosed/tested and onto treatment?

1. **HEALTH CARE UTILIZATION**

- Where do you usually go for health care? (probe government clinic, MRC/UVRI clinic, private doctor, healer) Why is that your preferred health care provider? How do you make a decision about where to go for care?
- If you make a choice NOT to go to the health center when you are feeling ill, what are the main reasons you choose not to go?
- If you make a choice to go to the health center when you are feeling ill, what are the main reasons that you make that choice?
- Think about the last time that you went to the (government or MRC/UVRI) health center... what were the best things about your visit?
- What were the hardest things about your visit?
- Did you feel like you received good care? Why/why not?
- Do you feel like you were treated well by health care staff? Why/why not?
- Did you feel embarrassed or like there were some things that it was not ok to talk to the health care center staff about? What things? Why were these difficult?
- How satisfied are you with the current health care providers at this health center? What makes you feel satisfied/dissatisfied?
- How satisfied are you with the facilities at this health center? What makes you feel satisfied/dissatisfied?
- Would you feel more comfortable going to the health center/talking about your ailments and symptoms if there was someone to talk to who knew more about older persons? What characteristics would you like that person to have? What would you like them to know about older persons?
